# Cumberland Infirmary

**Published:** 1920-12-11

**Authors:** 


					Cumberland Infirmary.
Barnes has been elected President of the
, n<J Infirmary, with which he has been officially
te?6nt ^?r ^orty-sovon years. He was presented at a
fathering of the honorary medical staff with a liand-
t}j . Ver inkstand, with a suitable inscription, as a mark
esteem and regard.

				

